# Supplementary material for: Exploring Pt-Pd Alloy Nanoparticle Cluster Formation through Conventional Sizing Techniques and Single-Particle Inductively Coupled Plasma—Sector Field Mass Spectrometry
Source: Nanomaterials (Basel). 2023 Sep 21;13(18):2610. doi: 10.3390/nano13182610 (PMC10537254; doi:10.3390/nano13182610)
Supplement: Supplementary file 1 [file nanomaterials-13-02610-s001.zip › nanomaterials-2593555-supplementary.pdf]

Exploring Pt-Pd Alloy Nanoparticle Cluster Formation through Conventional Sizing Techniques and Single-Particle Inductively Coupled Plasma—Sector Field Mass Spectrometry

Omar Martinez-Mora [1,2], Kristof Tirez [1], Filip Beutels [1], Wilfried Brusten [1], Luis F. Leon-Fernandez [1], Jan Fransaer [2], Xochitl Dominguez-Benetton [1] and Milica Velimirovic [1,*]

SUPPLEMENTARY INFORMATION

Pt_{75}-Pd_{25}

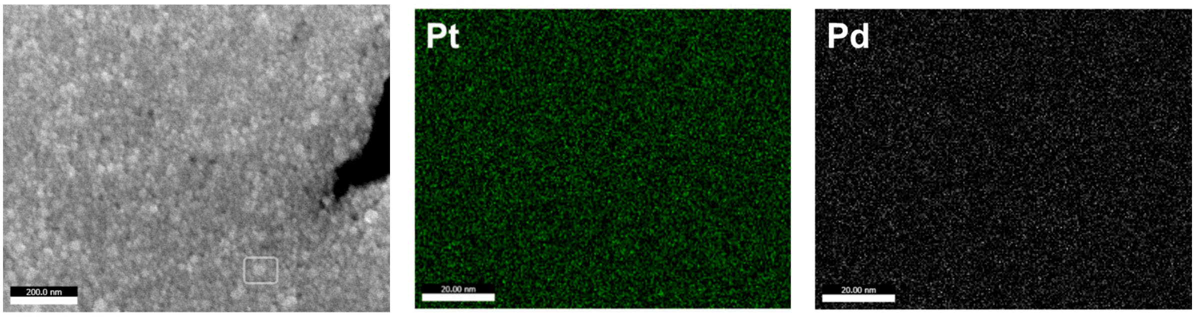

Figure S1. SEM-EDS mapping of Pt_{75}-Pd_{25}

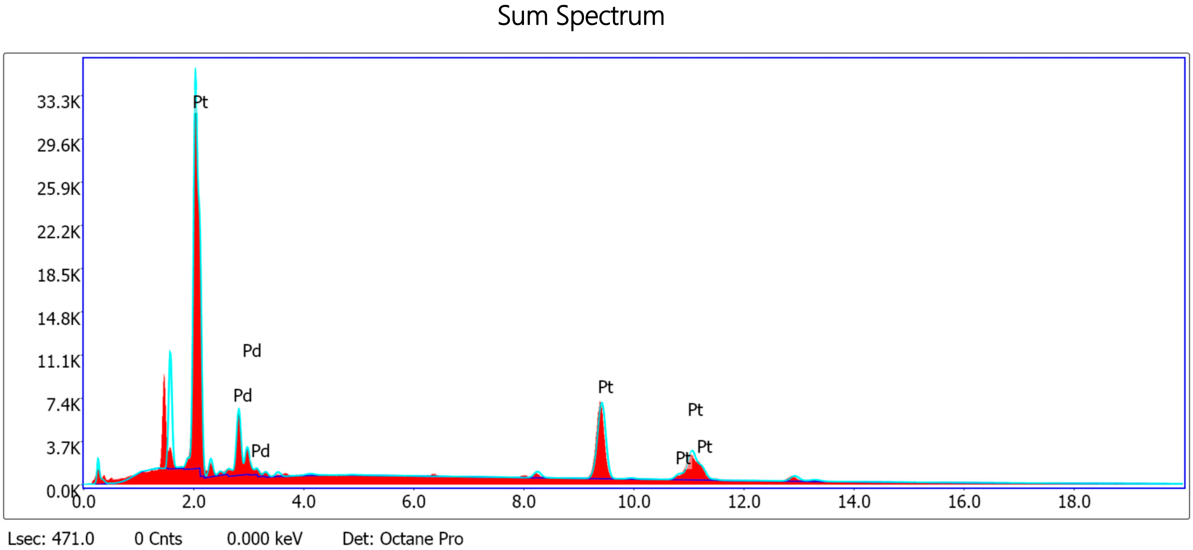

Figure S2. EDS spectrum of Pt_{75}-Pd_{25}

Pt_{50}-Pd_{50}

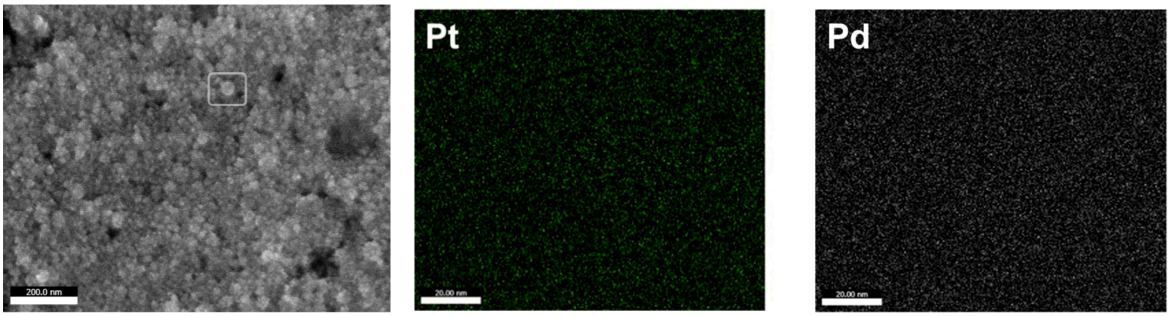

Figure S3. SEM-EDS mapping of Pt_{50}-Pd_{50}

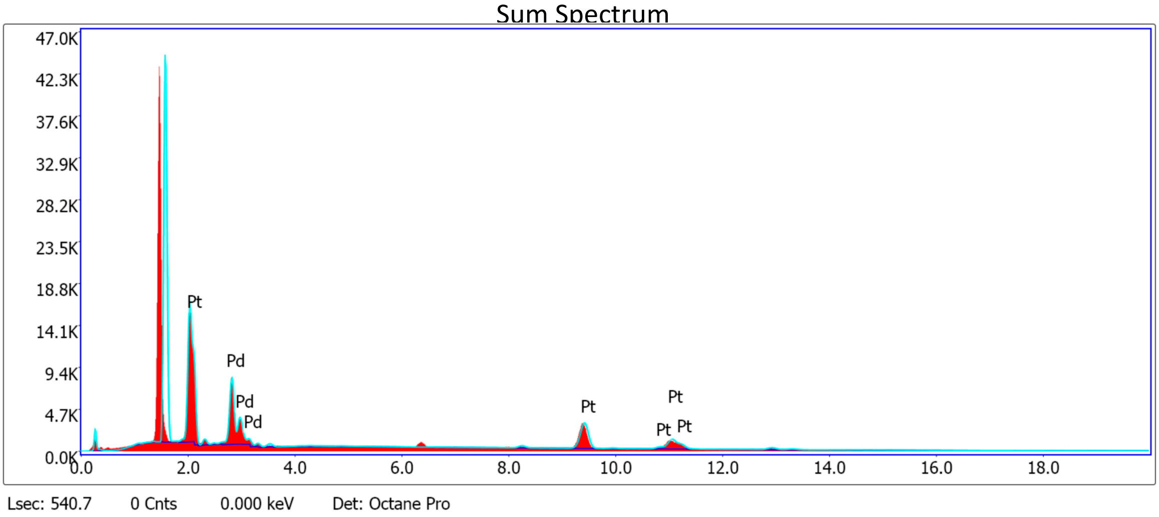

Figure S4. EDS spectrum of Pt_{50}-Pd_{50}

Pt_{25}-Pd_{75}

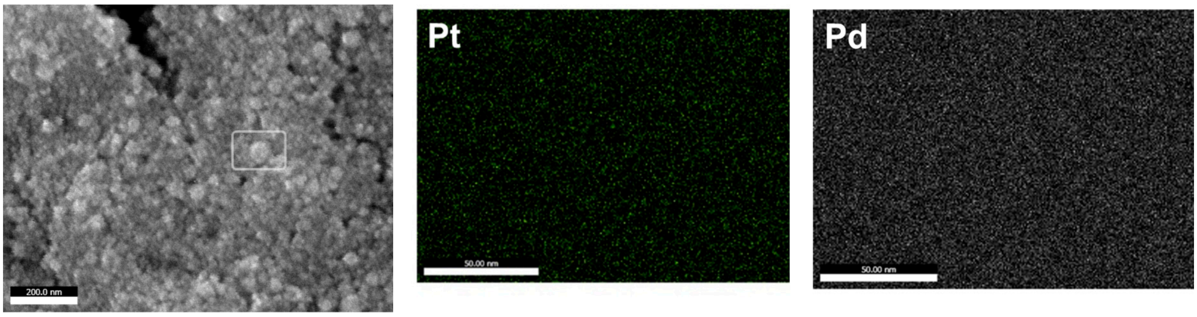

Figure S5. SEM-EDS mapping of Pt_{25}-Pd_{75}

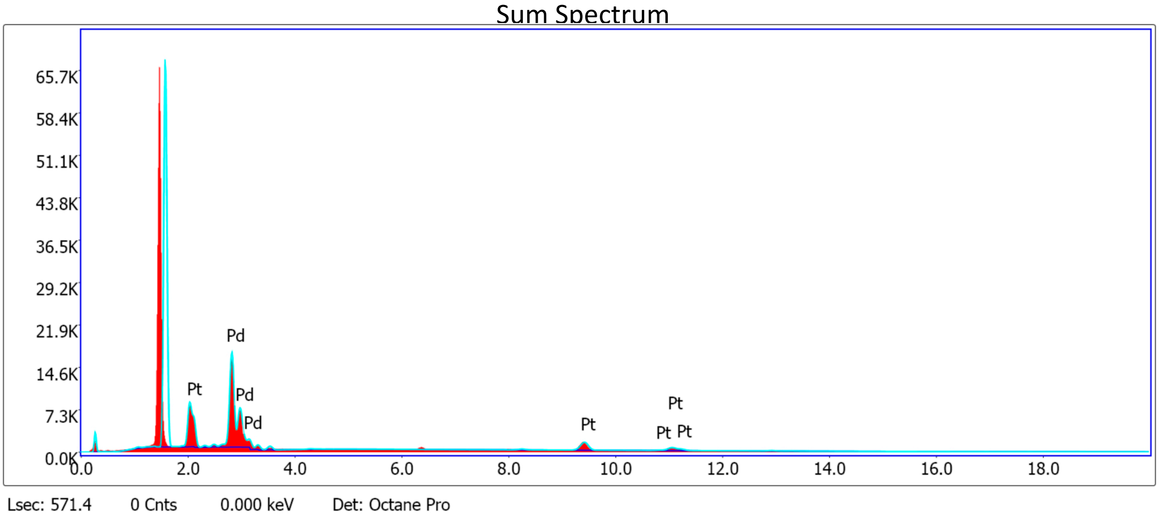

Figure S6. EDS spectrum of Pt_{25}-Pd_{75}

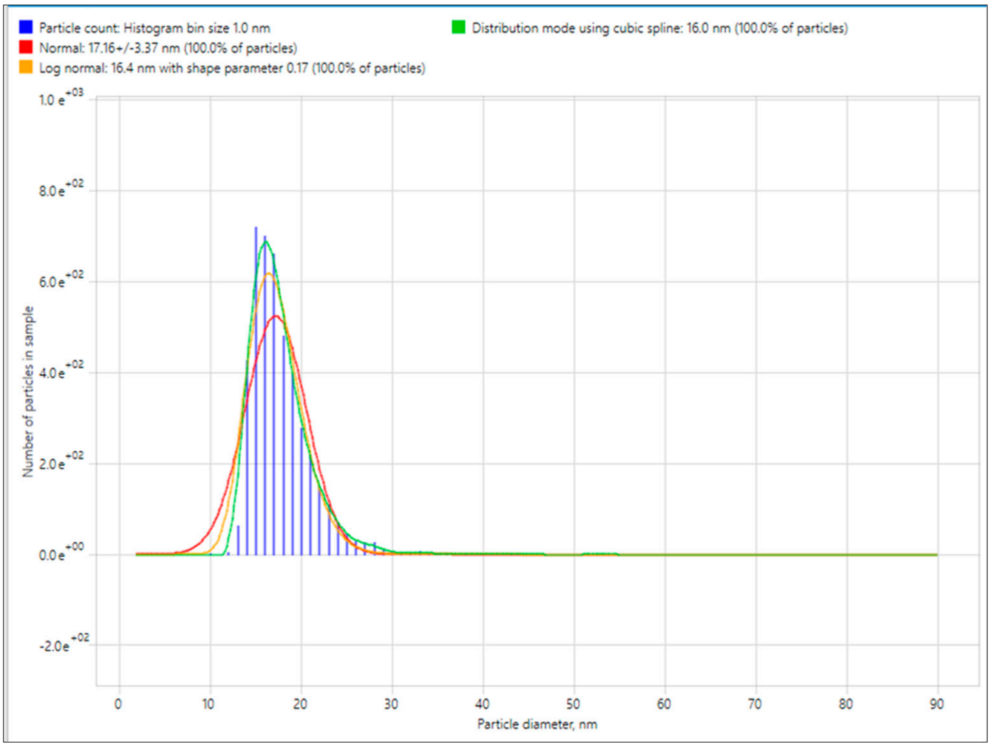

Figure S7. Particle size distribution of Pt (Pt100) NCs using different fitting models as obtained using spICP-SFMS.

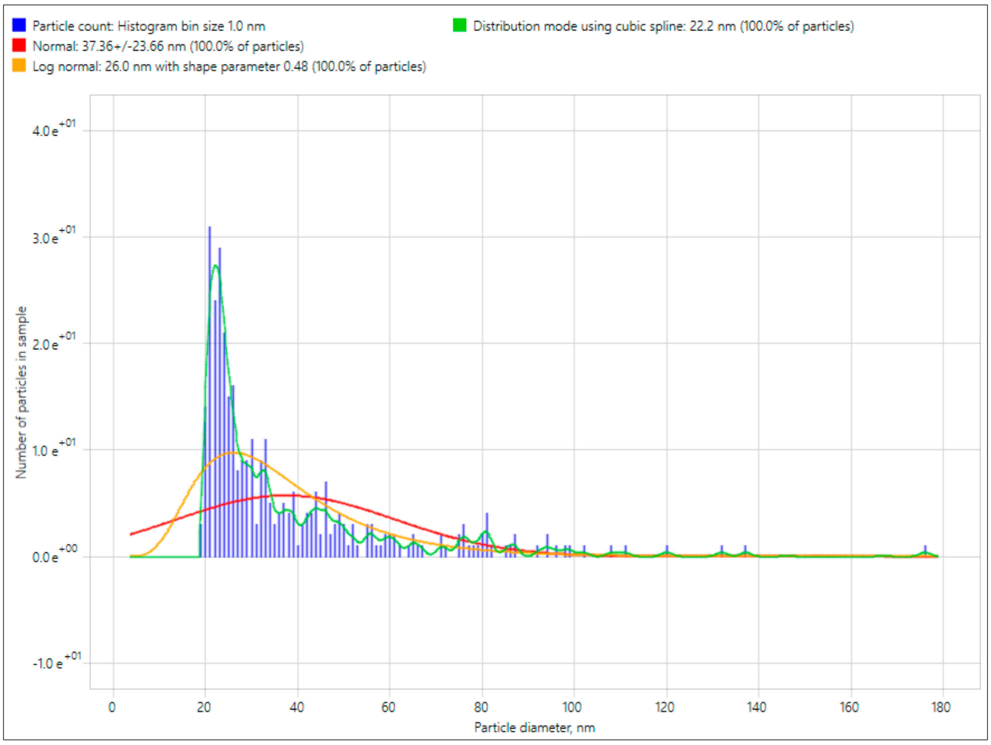

Figure S8. Particle size distribution of Pd (Pd100) NCs using different fitting models as obtained using spICP-SFMS.

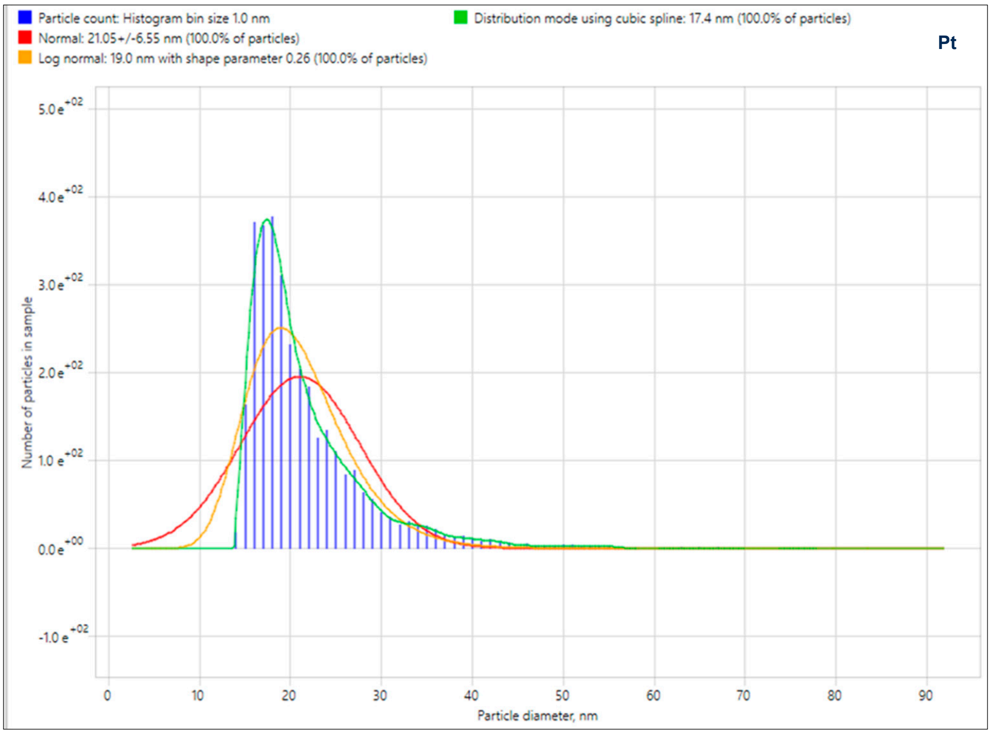

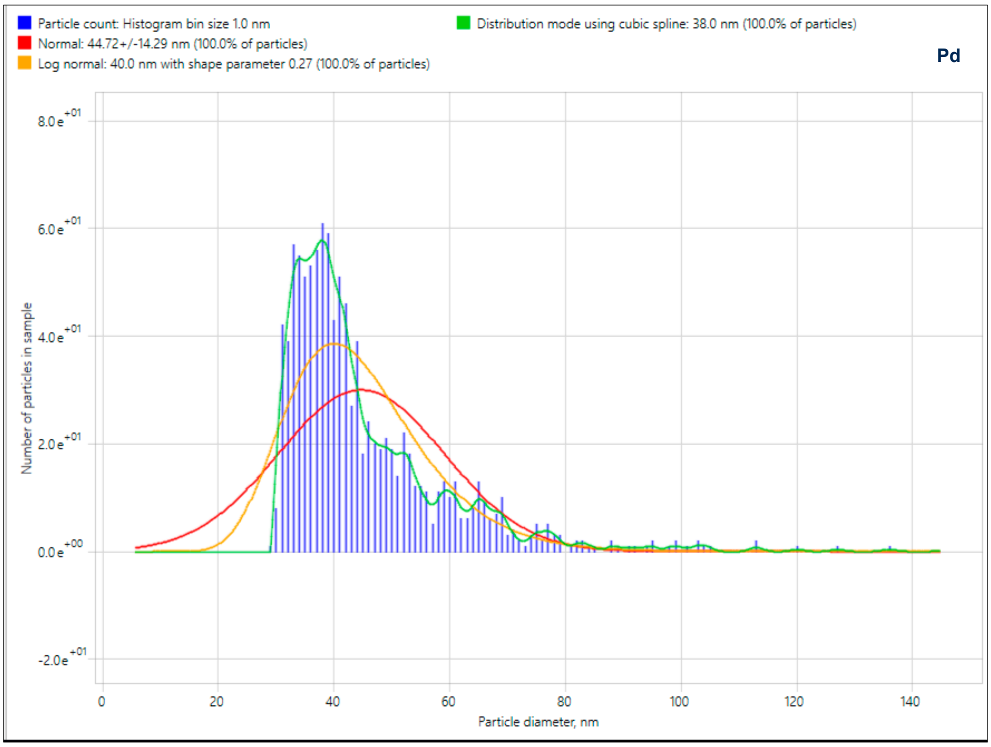

Figure S9. Particle size distribution of Pt and Pd using different fitting models as obtained using spICP-SFMS to calculate particle size distribution of Pt_{75}-Pd_{25} alloy NCs.

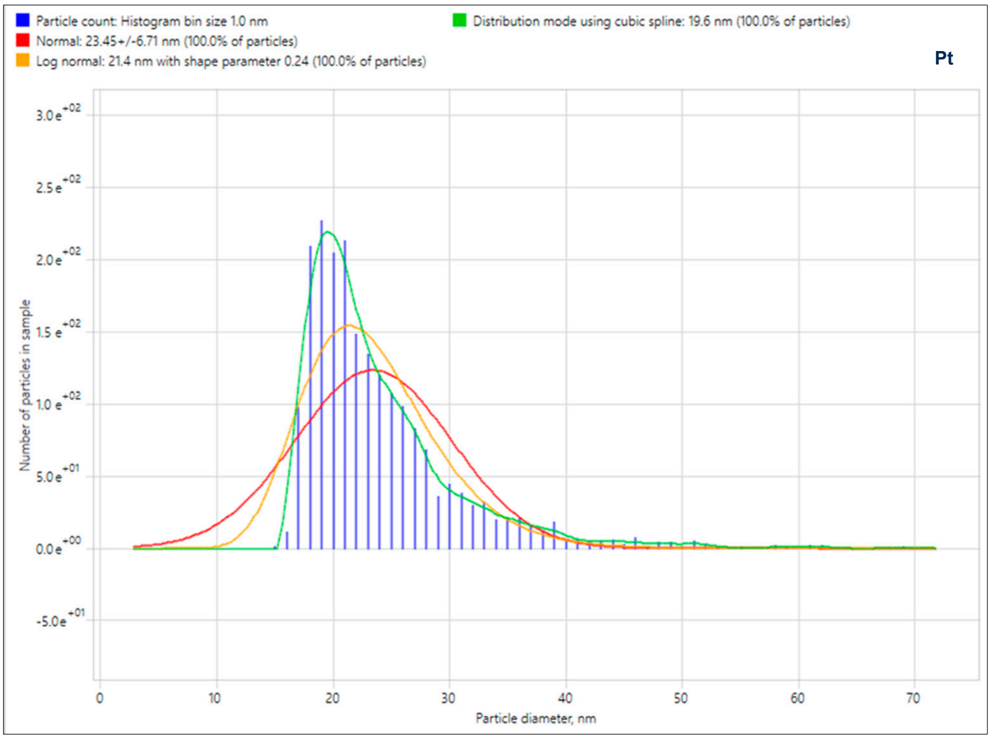

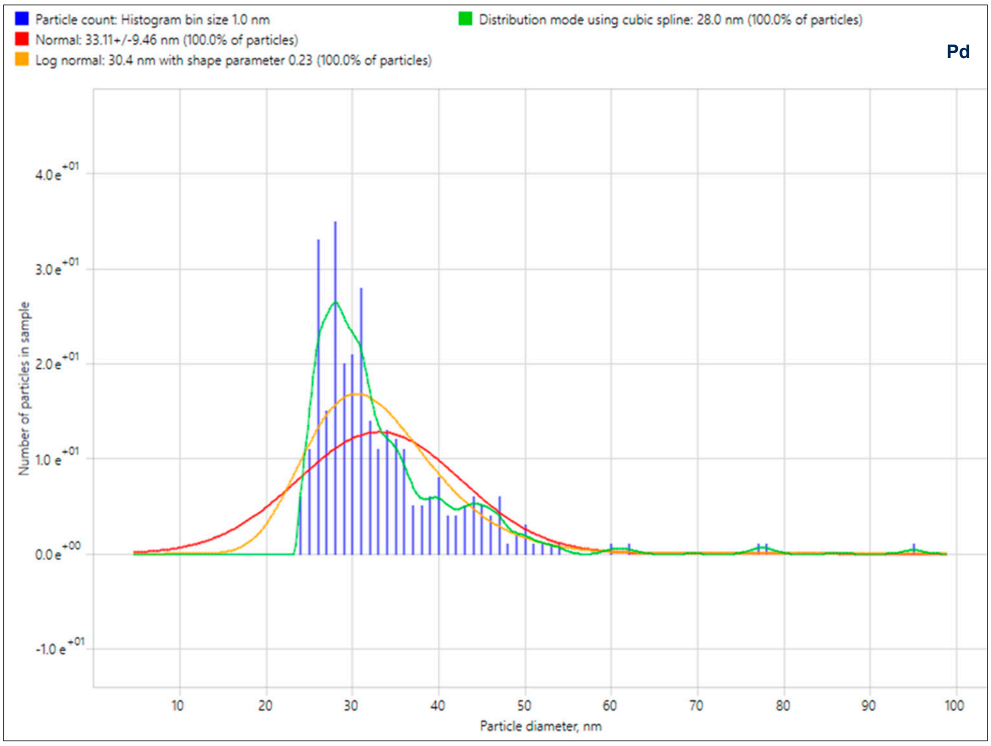

Figure S10. Particle size distribution of Pt and Pd using different fitting models as obtained using spICP-SFMS to calculate particle size distribution of Pt_{50}-Pd_{50} alloy NCs.

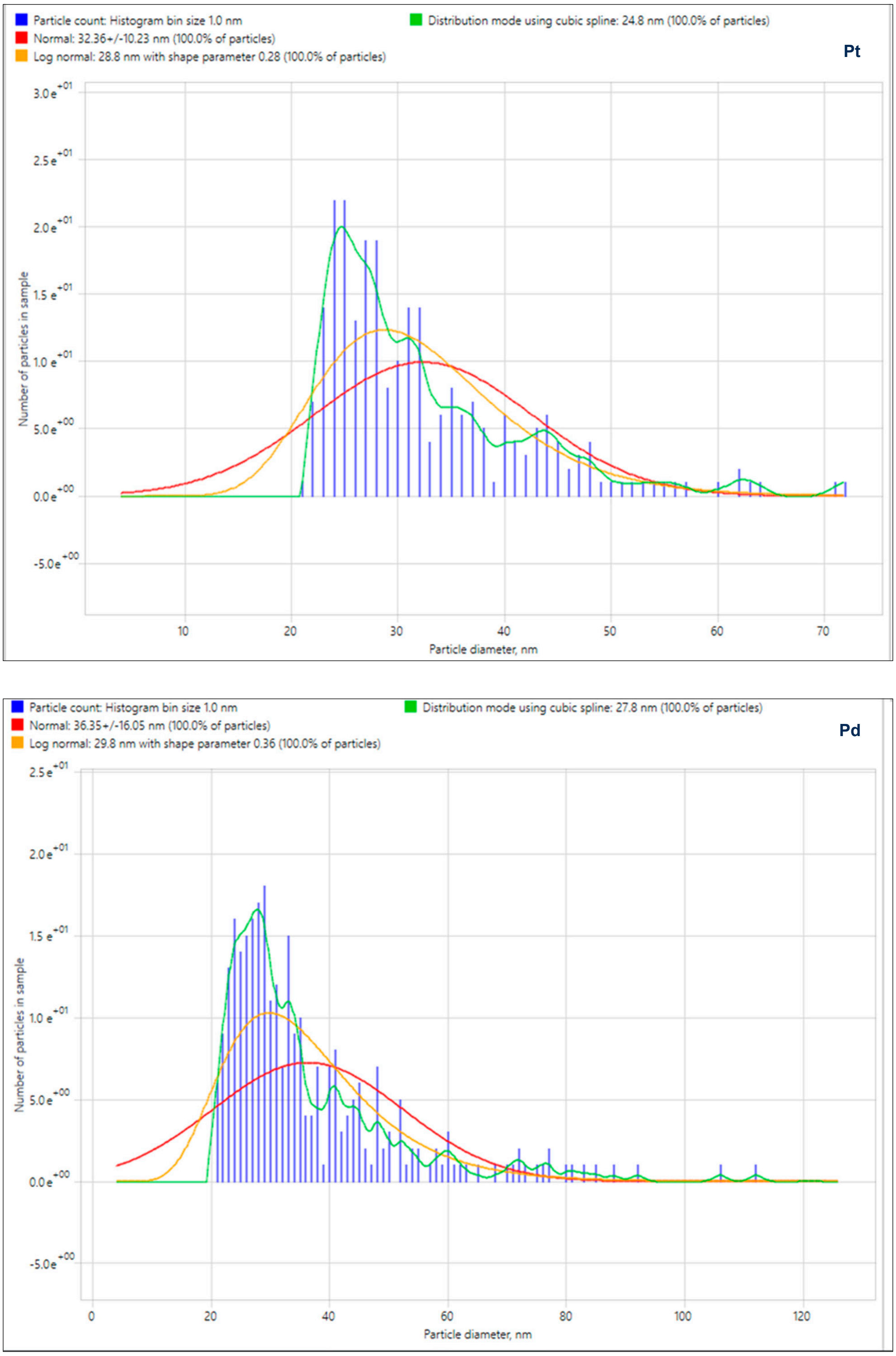

Figure S11. Particle size distribution of Pt and Pd using different fitting models as obtained using spICP-SFMS to calculate particle size distribution of Pt_{75}-Pd_{25} alloy NCs.
